# Parameter search of a CPG network using a genetic algorithm for a snake robot with tactile sensors moving on a soft floor

**DOI:** 10.3389/frobt.2023.1138019

**Published:** 2023-03-29

**Authors:** Hajime Tamura, Tetsushi Kamegawa

**Affiliations:** Graduate School of Interdisciplinary Science and Engineering in Health Systems, Okayama University, Okayama, Japan

**Keywords:** snake robot, tactile sensor, CPG network, soft floor, genetic algorithm

## Abstract

When a snake robot explores a collapsed house as a rescue robot, it needs to move through various obstacles, some of which may be made of soft materials, such as mattresses. In this study, we call mattress-like environment as a soft floor, which deforms when some force is added to it. We focused on the central pattern generator (CPG) network as a control for the snake robot to propel itself on the soft floor and constructed a CPG network that feeds back contact information between the robot and the floor. A genetic algorithm was used to determine the parameters of the CPG network suitable for the soft floor. To verify the obtained parameters, comparative simulations were conducted using the parameters obtained for the soft and hard floor, and the parameters were confirmed to be appropriate for each environment. By observing the difference in snake robot’s propulsion depending on the presence or absence of the tactile sensor feedback signal, we confirmed the effectiveness of the tactile sensor considered in the parameter search.

## 1 Introduction

Snake robots are engineering mimics of biological snakes. Despite their simple shape, biological snakes are able to move flexibly and adapt to various environments, such as poorly secured ground or narrow spaces. These characteristics of snakes suggest that snake robots could move through environments that are difficult for conventional mobile robots to traverse, and snake robots are expected to be applied to various tasks Hirose (1993).

Rescue robots are one application of snake robots. In the case of snake robot moving in a complex and narrow environment, it is necessary to adapt its shape to the environment by using tactile sensors. Snake robots equipped with tactile sensors across their bodies have been developed Liljebäck et al. (2012); Tadokoro (2019); Kano and Ishiguro (2020); Thandiackal et al. (2021). These snake robots are expected to be used for rescue activities in collapsed houses at disaster sites damaged by earthquakes and tsunamis as fast as possible and as long time as possible with as little energy consumption as possible without robot failure, thereby reducing secondary disasters. When a snake robot searches a collapsed house, it must overcome various obstacles including made of soft materials, such as futons and mattresses. For example, Micire (2008) conducted a mission to explore a collapsed building damaged by a hurricane with a robot and reported that a mattress in one bedroom became an obstacle to the robot’s movement. However, few studies have been conducted on robots that propel themselves over such mattress-like soft obstacles. We call mattress-like environment as a soft floor, which deforms when some force is added to it. The final goal of this study is to apply a snake robot to special and complex environments such as disaster sites. On disaster sites, rather than designing optimal control strategies for a snake robot in one specific environment, it is necessary to seek control strategies for a robot that can run in a variety of situations, including environments that have not been covered in previous research. One of the characteristics of the environment that has not been covered in previous research Liljebäck et al. (2013) is a soft floor. While there has been some research on mathematical models of soft environments. For example, literature Vogt et al. (2017) describes a soft tube environment, in this study it was necessary to construct a new soft planar model that interacts with a moving robot in a dynamics simulator. We propose the modeling method for a soft floor. In addition, we show that the control parameters of a snake robot moving on a hard floor are not necessarily optimal when the robot moves on a soft floor.

In this study, we focused on the central pattern generator (CPG) network as a control strategy for a snake robot to move on a soft floor. The CPG network is a control method for robots that applies a neural circuit believed to be involved in the periodic motion of living organisms Matsuoka (1987); Ijspeert (2008); Yu et al. (2014). Previous studies of CPG networks for snake robots include a study that constructed a CPG network that generates lateral undulation propulsion for a 2D snake robot Inoue et al. (2004). It is believed that by adding information from the outside world as feedback signals to the CPG network, environmentally adaptive behavior could be generated. For example, a study has been conducted in which using friction force as feedback signals Otaka et al. (2018). A study that generates undulatory swimming for a snake robot by CPG network with feedback information of force sensors has also been conducted Thandiackal et al. (2021). In this study, we constructed a new CPG network for the snake robot with tactile sensors that can move in three dimensions, which has been developed by our research group Tadokoro (2019). The constructed CPG network enabled the snake robot to move with sidewinding locomotion. However, it is difficult to find the appropriate parameters of the CPG network analytically because there are many parameters and they are related each other complex. In a previous study, Inoue et al. used the genetic algorithm (GA) Holland (1992) to find the parameters in a simple CPG network for their 2D snake robot performing lateral undulation propulsion Inoue et al. (2007). In this study, we should find the CPG parameters for 3D snake robot including feedback from tactile sensor contact with environment. The feedback from tactile sensor makes the CPG network more complex to find appropriate parameters. We have constructed a model of the snake robot moving on a soft floor in the simulation environment and found the CPG parameter. To verify the obtained parameters, comparative simulations were conducted using the parameters obtained for the soft and hard floor, and the parameters were confirmed to be appropriate for each environment. By observing the difference in snake robot’s propulsion depending on the presence or absence of the tactile sensor feedback signal, we confirmed the effectiveness of the tactile sensor considered in the parameter search.

## 2 A model of the snake robot and the environment

### 2.1 A model of the snake robot

A model of the snake robot was built on a simulator for the simulation. CoppeliaSim version 4.2.0 (Coppelia Robotics, Ltd., Switzerland) was used as the robot simulator, and the Open Dynamics Engine was used as the physics engine.

**FIGURE 1:**
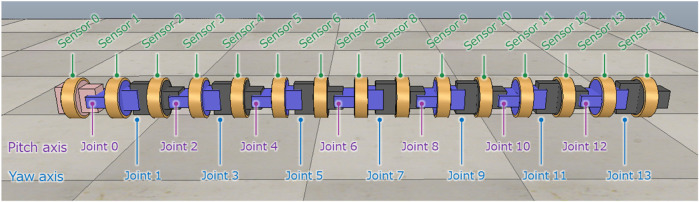
Simulation model of the snake robot.

Figure 1 shows the snake robot model built on the simulator. The snake robot has a total length of 1.35 m, a weight of 12.3 kg, a diameter of 0.09 m, a distance between joints of 0.09 m, 14 joints, and 15 tactile sensors. The diameter of the snake robot is the diameter of the outer circumference of the tactile sensor.

#### 2.1.1 Joint configuration of the snake robot

Snake robots are elongated robots with multiple active joints connected in series. In this study, the joint configuration is based on alternating connections of the pitch and yaw axes, which have been widely used in snake robots developed to date. The first joint is assumed to be a pitch-axis joint. The joints are numbered as “0, 1, 2, ⋯” starting from the first joint, and the joint numbered *i* is called joint *i*. The even-numbered joints are the pitch-axis joints, and the odd-numbered joints are the yaw-axis joints. The range of a joint is ±81.9 deg, this is due to contact of the adjacent links. The maximum torque is set as 8.4Nm and the maximum angular speed is set as 360.0 deg/*s*. *θ*_*i*_ is the target joint angle, and control to the target angle is controlled by the PID controller in the joint model of the robot simulator CoppeliaSim. In this study, the gains are set to *p* = 1.0, *I* = 0.0, and *D* = 0.0, making it a simple proportional control.

#### 2.1.2 Tactile sensor configuration of the snake robot

The tactile sensor model used in this study is a model of a center of pressure (CoP) sensor. A CoP sensor has a pressure-sensitive sheet on its circumference and can measure the position and magnitude of the applied force on the circumference Tadokoro (2019). Tactile sensors are attached to the center of each link to cover that link. When tactile sensors contact the external environment, the contact force can be obtained as a sensor value. The links are numbered as “0, 1, 2, ⋯” starting from the first link, and the link numbered *i* is called the link *i*. The tactile sensor attached to link *i* is called sensor *i*. The detail of the CoP sensor is described in the literature Tadokoro (2019). The simulation model in this study, it is modeled as having very high resolution which returns value of double precision floating point number.

**FIGURE 2:**
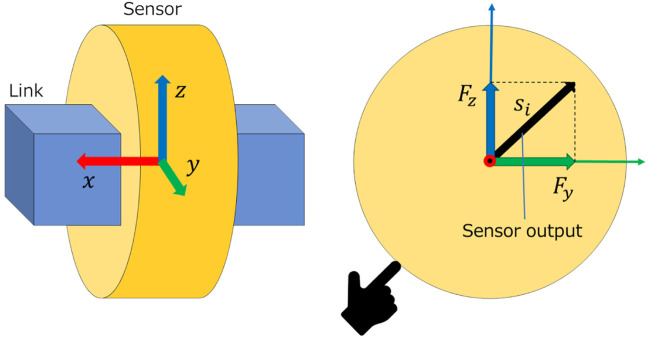
Model of tactile sensor.

The tactile sensor model was simulated using the model of the force sensor in CoppeliaSim. The coordinate of the force sensor is set as Figure 2 shows, and the components *F*_*y*_ and *F*_*z*_ of the force are obtained when contact occurs. $s_{i}=\sqrt{{F_{y}}^{2}+{F_{z}}^{2}}$ denotes the output *s*_*i*_ of sensor *i*. Note that the component of *F*_*x*_ is not used in this study.

### 2.2 Model of the soft floor

**FIGURE 3:**
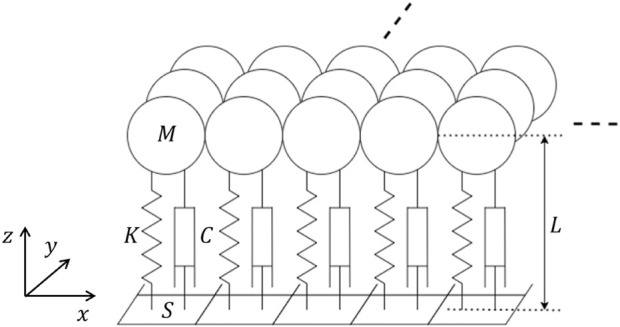
Soft floor model with a spring-mass-damper system.

In this study, the small units that make up a soft floor are modeled as a spring-mass-damper system. A conceptual diagram of the model is shown in Figure 3. Each block consists of one spring-mass-damper system. In our study, *L* is the thickness of the soft floor, *S* is the area of one block viewed from above, *M* is the mass of one block, *K* is the spring constant of one block, and *C* is the damping coefficient of one block.

The spring constant *K* of this model can be expressed using Young’s modulus *E* as follows:

$$K=\frac{S}{L}E.$$ (1)

The mass, spring, and damper system of one block satisfy the critical damping condition $C=2\sqrt{MK}$ so that it does not vibrate because this study focus on mattress-like soft obstacles as described in the introduction. *L* was set to a value sufficiently large for deformation volume that occurs when the snake robot is on a soft floor. *S* was set to a reasonable value considering the simulation accuracy and computation time. Although *S* should be as small as possible from the standpoint of simulation accuracy, too small *S* requires many blocks to support the snake robot, which increases the computation time. In this study, *S* was set to be as small as possible so that the simulation could be completed in realistic time. *M* was set to a value small enough relative to the mass of the snake robot. Specifically, we estimated the mass that a single block would have to support when the snake robot is placed on the soft floor in a straight line and set *M* to one-tenth of that mass. The reason for the sphere shape of the soft floor blocks is to eliminate the corners of the blocks and to avoid snagging between the snake robot and the soft floor.

**FIGURE 4:**
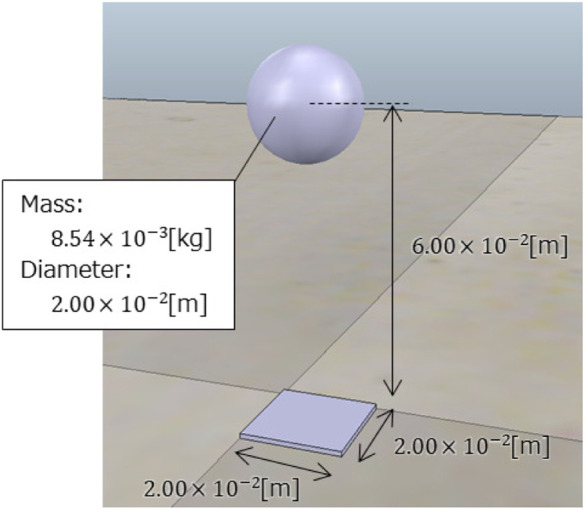
One block of the soft floor implemented in the simulator.

One block of the soft floor implemented in the simulator is shown in Figure 4. For implementation, the Young’s modulus of the soft floor was set to *E* = 8.75 × 10^3^Pa, referring to a mattress used in a paper on the mechanical properties of mattresses Inoue and Yamada (2016). Other parameters were as follows: *L* = 6.00 × 10^−2^ m, *S* = 4.00 × 10^−4^ m^2^, and *M* = 8.54 × 10^−3^kg. Note that the simulated part of the soft floor surface was spherical, with a 2.00 × 10^−2^ m diameter. The interaction between adjacent spheres is not considered in this study, and the soft floor is modeled as being deformed only in the vertical direction by a vertical downward force. In addition, the spheres have a set mass and are subjected to gravity in the dynamics simulator. The natural length of the spring is adjusted so that the thickness is 6.00 × 10^−2^ m when there is nothing on the sphere under gravity.

**FIGURE 5:**
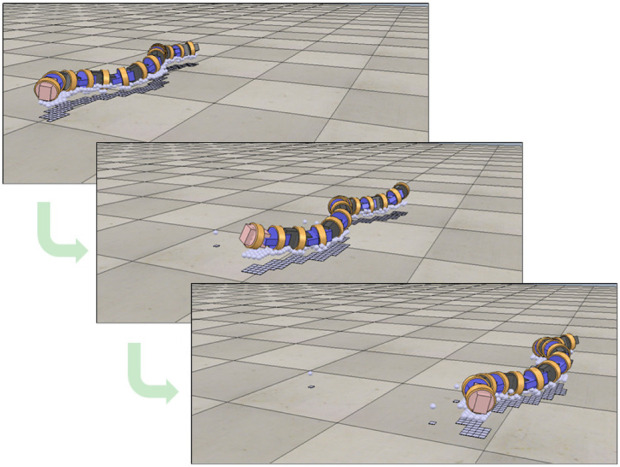
Generating the soft floor only in the vicinity of the snake robot.

The soft floor was modeled by arranging several of the single blocks shown in Figure 4 without gaps between them. Figure 5 shows the soft floor and the snake robot in the simulator. It can be seen that the blocks are clustered together to form the floor that supports the snake robot. The friction was set to 1.00 which is the default value of physics engine ODE to the snake robot and the soft floor surface, respectively. This allows the snake robot to be propelled almost without slipping on the soft floor surface as far as observing the behavior of the simulation.

## 3 CPG network

**FIGURE 6:**
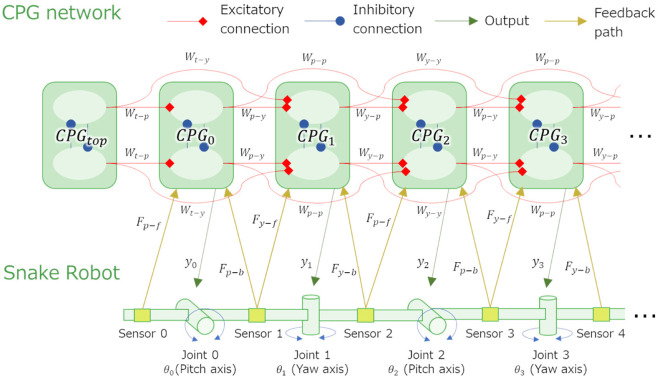
The central pattern generator (CPG) network in this study.

The CPG network constructed in this study is an extension of the CPG network that generates the sidewinding propulsion of a snake robot Tamura et al. (2020) and is shown in Figure 6.

Let *CPG*_*i*_ be the CPG model corresponding to joint *i* of the snake robot. There is only one CPG model that does not correspond to any joint, which is *CPG*_*top*_. The CPG model corresponding to the pitch-axis joint is called *CPG*_*pitch*_, and the CPG model corresponding to the yaw-axis joint is called *CPG*_*yaw*_.

**FIGURE 7:**
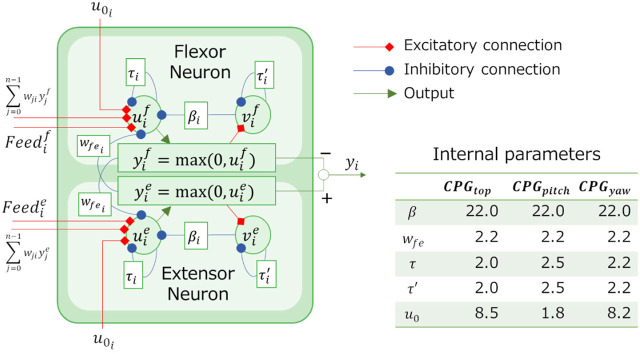
CPG model and its parameters.

For the CPG model in this study, Matsuoka’s model Matsuoka (1987) (Figure 7) is used, and the relationship between the output *y*_*i*_ of *CPG*_*i*_ and the angle *θ*_*i*_ of joint *i* is assumed to be *θ*_*i*_ = *y*_*i*_. The internal parameters *β*, *w*_*fe*_, *τ*, *τ*′, and *u*_0_, which are constant parameters determine the behavior of this CPG model.

The values of the internal parameters used are also shown in Figure 7. These values were obtained in preliminary study Tamura et al. (2020) that heuristically determine the parameters for the snake robot to be propel by sidewinding on a hard floor. Although *u*_0_ is originally given externally as an input from the upper center, it is classified as an internal parameter and as a constant in this study because it is not assumed that the behavior of the snake robot changes by the command from upper central nervous system.

The CPG network is formed by connecting the CPG models to each other. Each CPG model affects up to two CPG models ahead by excitatory connections. The connection weights, which determine the strength of the connections between CPGs, are *W*_*t*−*p*_ from *CPG*_*top*_ to *CPG*_*pitch*_, *W*_*t*−*y*_ from *CPG*_*top*_ to *CPG*_*yaw*_, *W*_*p*−*y*_ from *CPG*_*pitch*_ to *CPG*_*yaw*_, *W*_*p*−*p*_ from *CPG*_*pitch*_ to *CPG*_*pitch*_, *W*_*y*−*p*_ from *CPG*_*yaw*_ to *CPG*_*pitch*_, and *W*_*y*−*y*_ from *CPG*_*yaw*_ to *CPG*_*yaw*_. Each CPG model is also connected to tactile sensors attached to the links before and after the joint to which it corresponds. The feedback weights, which determine the strength of the feedback signal, are *F*_*p*−*f*_ and *F*_*y*−*f*_ for the front side and *F*_*p*−*b*_ and *F*_*y*−*b*_ for the back side for *CPG*_*pitch*_ and *CPG*_*yaw*_, respectively. Using these feedback weights, the feedback signals $Feed_{i}^{f}$ and $Feed_{i}^{e}$ of the CPG model are as follows:

$$Feed_{i}^{f}=Feed_{i}^{e}=F_{p-f}s_{i}+F_{p-b}s_{i+1}$$ (2)

where *s*_*i*_ is the output of sensor *i*. When $Feed_{i}^{f}$ equals to $Feed_{i}^{e}$ , a snake robot moves straight.

## 4 Parameter search using a GA

As usual, for this study the optimization problem of the CPG network which has a high number of parameters has been addressed using GA. The details are shown below.

### 4.1 Problem setting

**FIGURE 8:**
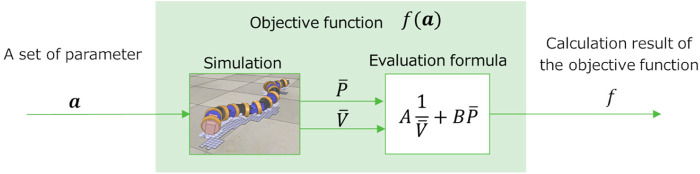
The method for calculating the objective function.

The parameter search problem is a minimization problem for an objective function *f*(***a***) whose argument is a vector ***a*** = (*a*_1_, *a*_2_, *a*_3_, … , *a*_*n*_) of parameters to be searched. In this study, the objective function is calculated using the results of moving a snake robot on a soft floor for a certain period of time in a simulator (Figure 8).

#### 4.1.1 Setting of search parameters

In this study, the search parameter ***a*** is the sequence of parameters of the CPG network. Because it is difficult to search for all parameters of the CPG network, we searched for only the connection weights and feedback weights of the CPG network parameter. In other words, ***a*** is a sequence of the parameters illustrated in Figure 6, which is expressed as follows:

$$a=\left(W_{t-p},W_{t-y},W_{p-y},W_{p-p},W_{y-p},W_{y-y},F_{p-f},F_{p-b},F_{y-f},F_{y-b}\right).$$ (3)

#### 4.1.2 Setting of the objective function

For a mobile snake robot used in rescue operation, it is important to increase the speed of movement. Thus, a propulsive motion with high speed can be regarded as a good motion. In addition, because battery consumption should be minimized when the snake robot moves, a propulsive motion with low power consumption can be regarded as a good motion. Accordingly, we define the objective function *f* in this study as follows.

$$f=A\frac{1}{\bar{V}}+B\bar{P},$$ (4)

$$\bar{V}=\frac{\left|d\right|}{T},$$ (5)

$$\bar{P}=\frac{W}{T},$$ (6)

**FIGURE 9:**
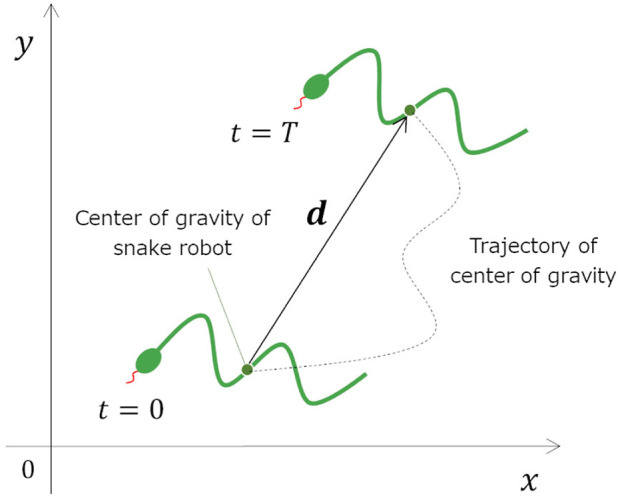
Definition of variables related to the snake robot’s movement.

Where $\bar{V}$ and $\bar{P}$ are the respective average movement speed and average power consumption of the snake robot when it is moved on a soft floor for a certain time, *A* and *B* are the weights of each term, *T* is the duration of time the robot moved on the soft floor, ***d*** is the displacement of the snake robot’s center of gravity between time 0 and *T* (see Figure 9), and *W* is the total power consumption of the snake robot between time 0 and *T*. It is assumed that we are going to conduct an experiment using a real snake robot which joints are driven by DC motor. We are going to measure voltage and current value of the DC motor in the experiment. In this study, we also use the voltage and estimate the current of the motor in the simulation as following. The total power consumption of the snake robot is defined as the sum of the power consumed by the motors that drive the joints of the snake robot, and is calculated using the following equations.

$$W=\overset{N-1}{\underset{i=0}{\sum }}\int_{0}^{T}|E_{v}I_{i}\left(t\right)|dt,$$ (7)

$$I_{i}=\lambda \tau_{i},$$ (8)

Where *N* is the number of snake robot joints, *E*_*v*_ is the power supply voltage, *I*_*i*_ is the current flowing in the motor of joint *i*, *τ*_*i*_ is the torque of joint *i*, and *λ* is the proportionality constant between the current and torque of the motor. *W* is calculated using the torque that can be obtained in the simulator according to Eq. 8. The values of *E*_*v*_ and *λ* were set to *E*_*v*_ = 12.0V and *λ* = 0.6548A/(N ⋅m), following the specifications of the motor used in the actual snake robot owned by our laboratory.

### 4.2 Setup of the GA

Because this study considers the problem of minimizing the objective function, reversing the magnitude of the objective function and the fitness of the GA is necessary. Thus, the fitness of the GA was set to *fitness* = −*f* using the objective function *f*.

This study used a genotype in which the search parameters defined in Eq. 3 are expressed as a bit string and are arranged in a sequence. Each parameter was set to 12 bits, with *W*_*t*−*p*_, *W*_*t*−*y*_, *W*_*p*−*y*_, *W*_*p*−*p*_, *W*_*y*−*p*_, and *W*_*y*−*y*_ as values between 0.00 and 2.00 and *F*_*p*−*f*_, *F*_*p*−*b*_, *F*_*y*−*f*_, and *F*_*y*−*b*_ as values between −0.20 and 0.20. The range of parameters are decided according to a preliminary study so that the robot can move by keeping periodical motion.

In this study, ranking selection was used for selection to speed up the convergence of GA solutions; uniform crossover, for crossover; and simple mutation, for mutation as the genetic manipulation of the GA. The details of the ranking selection used in this study are described below. The best individual is ranked as the 0th, followed by the first, second, and so on in order of fitness. Let *p*_*k*_ be the selection probability of the *k*th individual. A truncated rank *k*_*t*_ is defined so that the selection probability of individuals with a rank lower than *k*_*t*_ is zero. Subsequently, the selection probabilities of individuals from the 0th to the *k*th ranks are decreased at a constant rate.

## 5 Simulation

### 5.1 Simulation of parameter search using the GA

We conducted simulations to determine the parameters of the CPG network using the GA described in Chapter 4. The snake robot was operated for a certain period of time on a soft floor and a hard floor, successively, and the objective function *f* was calculated. In order to determine the parameters for hard and soft floors, the hard floor was implemented also in the simulation environment. The hard floor was realized without the vertical movement of the spring and damper described in Section 2.2. The search on the soft floor was referred to as the soft floor condition, the search on the hard floor was referred to as the hard floor condition, and the parameters were obtained using a GA for each condition.

**FIGURE 10:**
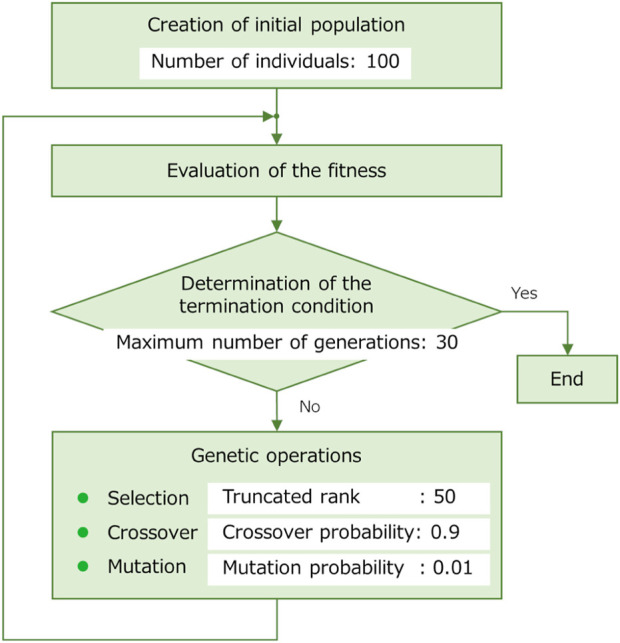
The flow and parameters of the GA.

The parameters included in the objective function equations (Eqs. 4–6) are as follows: the weight of the average speed term, *A* = 100; the weight of the average power consumption term, *B* = 1; and the robot’s operating time, *T* = 30s. The weight A and B was decided according to a preliminary study. In the preliminary study, the snake robot moved in typical sidewinding motion on the soft floor, and its average movement speed and average power consumption were recorded. Based on the recorded value, the weight of A and B were adjusted so that the terms were of similar magnitude. The weights A and B are adjusted according to a robot’s application. For example, if power consumption or actuator load is more important than travel speed, the value of B can be adjusted to a larger value. The flow and parameters of the GA are shown in Figure 10. The elitism was adopted in the selection of genetic operations.

**FIGURE 11:**
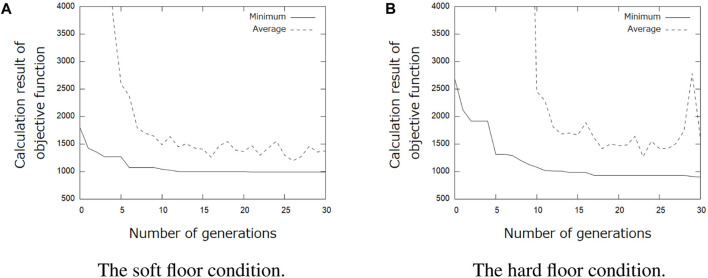
Change of objective function value during genetic algorithm (GA) execution.

**FIGURE 12:**
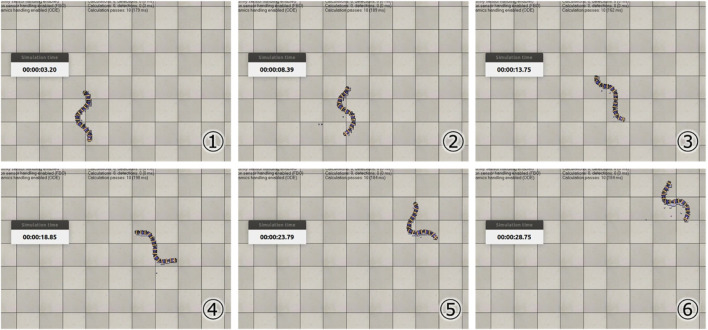
Propulsion behavior on the soft floor with parameters obtained under the soft floor condition.

**FIGURE 13:**
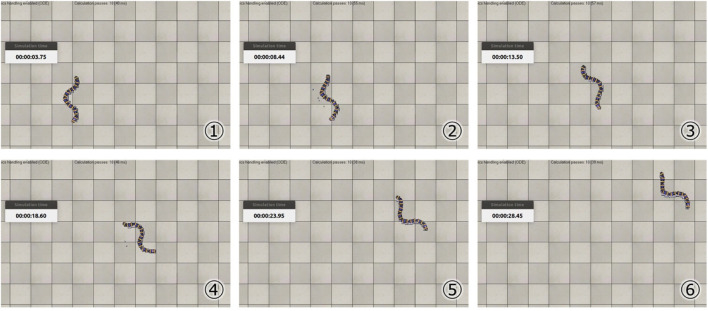
Propulsion behavior on the hard floor with parameters obtained under the hard floor condition.

Table 1 shows the results of the parameter search using the GA. It can be seen that different parameters were obtained when searching under the soft and hard floor conditions. The feedback weights tend to be smaller on a hard floor than on a soft floor. This is because the feedback signal from the tactile sensor tends to increase momentarily on hard floors due to collisions between the robot and the floor, and the weights are adjusted to reduce the effect of such collisions. Figure 11 shows the change in calculation result of the objective function for each number of generations during GA execution. In addition, Figure 12 and 13 show how the snake robot was propelled with the parameters obtained from the GA. In both conditions, the snake robot remains the starting point with the parameters before the search. However, with the parameters after the search, the snake robot propelled efficiently by sidewinding propulsion in each condition as shown in Figure 12 and 13.

**TABLE 1:** Parameters obtained using the GA.

Parameters	The soft floor condition	The hard floor condition
*W*_*t*−*p*_	0.553	0.912
*W*_*t*−*y*_	0.0215	0.0518
*W*_*p*−*y*_	0.0694	0.0186
*W*_*p*−*p*_	1.30	0.871
*W*_*y*−*p*_	0.752	1.40
*W*_*y*−*y*_	1.32	0.915
*F*_*p*−*f*_	0.0184	0.00650
*F*_*p*−*b*_	0.0608	0.0426
*F*_*y*−*f*_	−0.182	0.0171
*F*_*y*−*b*_	0.162	0.0185

### 5.2 Simulations to validate the obtained parameters

**FIGURE 14:**
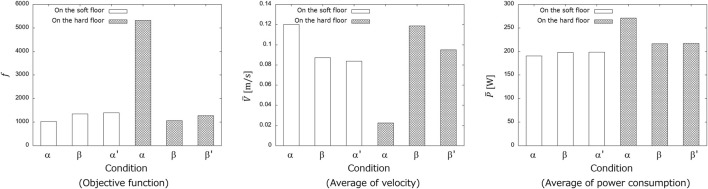
Results of propulsion experiment. Condition α, β, α′ and β′ are in parameters obtained under the soft floor condition, obtained under the hard floor condition, obtained under the soft floor condition when feedback was cut off, obtained under the hard floor condition when feedback was cut off, respectively.

The parameters obtained for the soft and hard floor conditions were used to compare the propulsion performance on the soft and hard floors, respectively. To confirm the effect of the feedback signal, a simulation in which the snake robot was moved by cutting off the feedback signal was conducted. The objective function in Eq. 4 was used as a measure of propulsion performance. Simulations were conducted five times under each condition, and the averages were compared. Figure 14 shows the results of the simulation. Each graph compares the value of the objective function *f*, the robot’s average velocity $\bar{V}$ and average of power consumption $\bar{P}$ when the snake robot moves on the soft floor and hard floor with the obtained parameters. For each graph, smaller *f* is better, larger $\bar{V}$ is better, and smaller $\bar{P}$ is better. On the soft floor, the movement by the parameters under the soft floor condition reduced the objective function value. Similarly, on the hard floor, the movement by the parameters under the hard floor conditions reduced the objective function value. When the feedback signal was cut off, the objective function values were larger than those when the feedback signal was present on both the soft and hard floors, indicating that the snake robot was unable to move appropriately because of the loss of tactile sensor feedback.

### 5.3 Discussion

We assume that the snake robot began sidewinding propulsion with the obtained parameters because the parameters of the preliminary study were used for the internal parameters of the CPG model. If the internal parameters of the CPG model can also be searched using the GA in future studies, a different propulsion behavior from the one observed in this study could be generated.

The results in Figure 14 showed that the appropriate parameters for the CPG network vary depending on the environment and that the GA could search for suitable parameters for the soft floor. In addition, it was confirmed that propulsion efficiency changes depending on the presence or absence of feedback signals, indicating that the GA can search for parameters that consider the effect of feedback signals. These results showed that the appropriate propulsion of a snake robot depends not only on the robot’s internal parameters but also on the interaction between the robot and the environment.

## 6 Conclusion

In this study, we constructed a CPG network for a snake robot equipped with tactile sensors and searched for parameters to propel the snake robot on the soft floor. The models of the snake robot and the soft floor were simulated so that the objective function in the parameter search could be calculated based on the simulation results. To use the GA for parameter search, fitness, genotype, and genetic manipulation were considered. Parameter search simulations were conducted using the constructed software in two different environments, a soft floor and a hard floor, and behaviors of the snake robot were compared using the obtained parameters. As a result of the simulation, the parameters of the CPG network suitable for the soft floor were obtained.

One of the future tasks is to investigate a method to adaptively operate the snake robot on floors with different softness and friction. Although this study explored the parameters of the CPG network considering the feedback signals from the sensors, it was for the specific given environment. CPG-based gait generation is expected to allow a snake robot to adapt to environments and move while updating internal parameters in real time, even during periodic movements. We believe that by developing an objective function that considers the different softness and different coefficient of friction of the floor, the snake robot will be able to adaptively move to the floor. Another future task includes searching for the internal parameters of the CPG model, which were not searched in this study, to generate propulsive motions that are more suitable for soft floors. By the further work, we expect that a different propulsive motion from sidewinding propulsion, which is the motion obtained in this study, will emerge. In addition, the proposed method will be implemented to a real snake robot and should be verified by conducting experiments. Ultimately, a snake robot will be used as a rescue robot in real-world complex environments.

## Data Availability

The datasets generated and/or analyzed during the current study are available from the corresponding author on reasonable request.

## Appendix A

Details of the CPG model used in this study are described below. The CPG model used in this study is Matsuoka’s CPG model (Figure 7). The output of the CPG model is the difference between the outputs of the extensor and flexor neurons. The dynamics of the CPG model can be expressed mathematically by Eq. 9.

$$\left\{\begin{array}{cc} & \tau_{i}{\dot{u}}_{i}^{\left\{f,e\right\}}+u_{i}^{\left\{f,e\right\}}=\overset{n}{\underset{j=1}{\sum }}w_{ji}y_{j}^{\left\{f,e\right\}}-w_{fe_{i}}y_{i}^{\left\{e,f\right\}}-\beta_{i}v_{i}^{\left\{f,e\right\}}+u_{0_{i}}+Feed_{i}^{\left\{f,e\right\}}\\ & y_{i}^{\left\{f,e\right\}}=\max\left(0,u_{i}^{\left\{f,e\right\}}\right)\\ & \tau_{i}^{\prime }{\dot{v}}_{i}^{\left\{f,e\right\}}+v_{i}^{\left\{f,e\right\}}=y_{i}^{\left\{f,e\right\}}\\ & y_{i}=y_{i}^{e}-y_{i}^{f} \end{array}\right.$$
(9)

where the subscripts *e* and *f* denote flexor and extensor neurons, respectively, and the subscripts *i* and *j* denote those of the *i*th or *j*th CPG. $u_{i}^{\left\{f,e\right\}}$ is the state of membrane potential of a CPG neuron, $v_{i}^{\left\{f,e\right\}}$ is the state of autoinhibition of a CPG neuron, $y_{i}^{\left\{f,e\right\}}$ is the output of the CPG neuron, $u_{0_{i}}$ is the driving input from the upper neuron of the CPG, *w*_*fe*_ is the mutual inhibition coefficient between extensor and flexor neurons, *y*_*i*_ is the output of the CPG, $y_{j}^{\left\{f,e\right\}}$ is the input from the other CPG, *n* is the number of CPG model, *w*_*ji*_ is the connection weight from *j*th to *i*th CPG model $Feed_{i}^{\left\{f,e\right\}}$ is the feedback signal from the sensor.
